# Survival Outcomes of Open Versus Robot-Assisted Radical Cystectomy: A Large-Scale Multicenter Propensity Score Matched Study

**DOI:** 10.3390/jcm15093559

**Published:** 2026-05-06

**Authors:** Jong Ho Park, Sangchul Lee, Seung-Hwan Jeong, Ja Hyeon Ku, Kyung Hwan Kim, Jong Kil Nam, Bumjin Lim, BumSik Hong, Wook Nam, Sung Gu Kang, Seok Ho Kang, Tae Gyun Kwon, Tae-Hwan Kim, Jieun Heo, Won Sik Ham, Geehyun Song, Ho Kyung Seo, Wan Song, Hyun Hwan Sung, Byong Chang Jeong, Jong Jin Oh

**Affiliations:** 1 Department of Urology, Seoul National University Bundang Hospital, Seongnam 13620, Republic of Korea; qltyq0627@gmail.com (J.H.P.); uromedi@naver.com (S.L.); 2 Department of Urology, Seoul National University College of Medicine, Seoul 03080, Republic of Korea; 3 Department of Urology, Seoul National University Hospital, Seoul 03080, Republic of Korea; 11shjeong@snu.ac.kr (S.-H.J.); kuuro70@snu.ac.kr (J.H.K.); 4 Department of Urology, Pusan National University Hospital, Busan 49241, Republic of Korea; bravekim80@naver.com; 5 Department of Urology, Pusan National University Yangsan Hospital, Yangsan 50612, Republic of Korea; tuff-kil@hanmail.net; 6 Department of Urology, Asan Medical Center, University of Ulsan College of Medicin, Seoul 05505, Republic of Korea; lbj1986@hanmail.net (B.L.); bshong@amc.seoul.kr (B.H.); 7 Department of Urology, Gangneung Asan Hospital, University of Ulsan College of Medicine, Gangneung 25440, Republic of Korea; wooki6258@naver.com; 8 Department of Urology, Korea University Anam Hospital, Korea University Medical Center, Korea University School of Medicine, Seoul 02841, Republic of Korea; kkangsung76@daum.net (S.G.K.); mdksh@korea.ac.kr (S.H.K.); 9 Department of Urology, Kyungpook National University School of Medicine, Daegu 41944, Republic of Korea; tgkwon@knu.ac.kr (T.G.K.); doctork@knu.ac.kr (T.-H.K.); 10 Department of Urology and Urological Science Institute, Yonsei University College of Medicine, Seoul 03722, Republic of Korea; heoji87@yuhs.ac (J.H.); uroham@yuhs.ac (W.S.H.); 11 Department of Urology, Center for Urologic Cancer, National Cancer Center, Goyang 10408, Republic of Korea; geehyuns@gmail.com (G.S.); seohk@ncc.re.kr (H.K.S.); 12 Department of Urology, Samsung Medical Center, Sungkyunkwan University School of Medicine, Seoul 06351, Republic of Korea; wan.song@samsung.com (W.S.); hyunhwan.sung@samsung.com (H.H.S.); bc2.jung@samsung.com (B.C.J.)

**Keywords:** urinary bladder neoplasms, cystectomy, robotic surgical procedures, survival analysis, propensity score

## Abstract

**Background/Objectives**: While robot-assisted radical cystectomy (RARC) is widely adopted, its long-term survival impact relative to open radical cystectomy (ORC) remains uncertain. We compared survival outcomes between ORC and RARC using a propensity score-matched multicenter cohort. **Methods**: We retrospectively analyzed 3972 radical cystectomy patients from 11 Korean tertiary centers between 2003 and 2024. After stratifying by surgical approach (ORC vs. RARC), 1:1 propensity score matching (PSM) mitigated baseline imbalances. Overall survival (OS), cancer-specific survival (CSS), and recurrence-free survival (RFS) were evaluated using Kaplan–Meier and multivariable Cox analyses. **Results**: PSM yielded 473 well-balanced patients per group. Compared to ORC, RARC was associated with a longer operative time but demonstrated a superior perioperative profile, including reduced estimated blood loss, lower intraoperative transfusion rates, shorter hospital stays, higher lymph node yields, and lower positive margin rates. RARC significantly improved OS (5-year: 75.4% vs. 56.1%; 10-year: 68.3% vs. 44.5%; *p* < 0.001) and CSS (5-year: 88.1% vs. 71.6%; 10-year: 82.7% vs. 67.7%; *p* < 0.001), with comparable RFS (5-year: 59.6% vs. 54.8%; 10-year: 51.1% vs. 47.4%; *p* = 0.155). Multivariable analyses confirmed RARC as an independent predictor of improved OS (hazard ratio [HR] 0.564, *p* < 0.001) and CSS (HR 0.474, *p* < 0.001). **Conclusions**: RARC demonstrated superior perioperative outcomes and favorable survival trends compared to ORC, with no difference in RFS. Although RARC appears to be an oncologically safe alternative, these exploratory survival benefits require cautious interpretation due to potential residual confounding. Further prospective validation is warranted.

## 1. Introduction

Bladder cancer (BC) is one of the most prevalent malignancies worldwide, and the second most common genitourinary malignancy [1]. Radical cystectomy (RC) with pelvic lymph node dissection (PLND) remains the gold standard for treating muscle-invasive and high-risk non-muscle-invasive BC [2,3]. Despite its oncologic efficacy, RC is a highly complex operation associated with considerable perioperative morbidity [4,5].

Over the past decade, the adoption of robot-assisted radical cystectomy (RARC) has steadily increased, supported by accumulating evidence demonstrating its oncologic safety and perioperative advantages over open radical cystectomy (ORC) [6,7,8,9,10,11,12]. While these perioperative benefits are generally well recognized, the impact of RARC on survival outcomes relative to ORC remains controversial, with prior studies reporting inconsistent results [13,14,15,16,17,18].

The RAZOR trial [18] demonstrated the non-inferiority of RARC to ORC regarding 2-year progression-free survival, yet it lacked long-term survival data. Moreover, many previous studies investigating the oncologic outcomes of ORC and RARC [3,5,7,8,14,17] relied on patients treated in earlier surgical eras and thereby limiting their applicability to current practice. Consequently, contemporary real-world evidence regarding long-term survival outcomes remains limited.

In this study, we evaluated a large-scale, multicenter cohort of patients who underwent ORC or RARC for BC and compared survival outcomes using propensity score matching.

## 2. Materials and Methods

### 2.1. Study Population

We retrospectively analyzed the medical records of 3972 patients who underwent RC for BC at 11 tertiary medical centers in South Korea between October 2003 and January 2024. Eligibility was limited to patients with pathologically confirmed urothelial carcinoma who received RC with PLND. We excluded those with distant metastasis, incomplete clinical data, or concurrent malignancies other than upper tract urothelial carcinoma (UTUC). The cohort was then stratified into two groups based on the surgical approach: ORC or RARC.

### 2.2. Variables and Outcomes

Baseline characteristics included age, sex, body mass index (BMI), diabetes mellitus (DM), hypertension (HTN), American Society of Anesthesiologists (ASA) physical status, clinical TNM stage, presence of concurrent UTUC, and neoadjuvant chemotherapy (CTx) status. Perioperative variables were date of surgery, operation type, urinary diversion type, estimated blood loss (EBL), intraoperative transfusion status, operative time, length of hospital stay (LOS), and 90-day readmission. Pathological outcomes comprised LN yield, pathological TNM stage, tumor grade, soft tissue surgical margin (STSM) status, presence of concurrent carcinoma in situ (CIS), presence of lymphovascular invasion (LVI), as well as adjuvant CTx status. Oncologic outcomes, such as recurrence and mortality, were documented as well. Tumor grading and staging were determined according to the 2004 World Health Organization/International Society of Urologic Pathology consensus and the 2017 American Joint Committee on Cancer classifications, respectively.

### 2.3. Statistical Analyses

To compare continuous data, we first verified normality via the Shapiro–Wilk test. Subsequently, *t*-tests or Wilcoxon rank-sum tests were performed, while categorical variables were analyzed using Fisher’s exact or Chi-square tests. Descriptive statistics for continuous parameters are presented as means ± standard deviations (SDs) or as medians with interquartile ranges (IQRs), according to their distribution. Frequencies and percentages were used to summarize categorical information.

**Figure 1:**
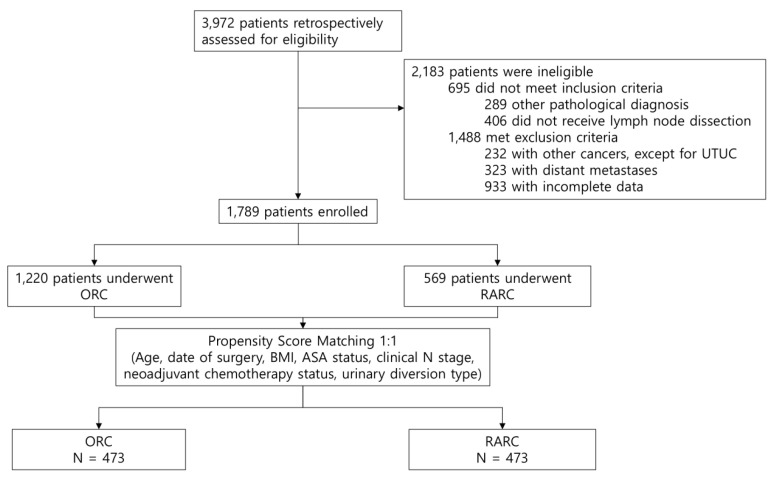
Study flow diagram. UTUC, upper tract urothelial carcinoma; ORC, open radical cystectomy; RARC, robot-assisted radical cystectomy; BMI, body mass index; ASA, American Society of Anesthesiologists.

To mitigate selection bias and balance baseline characteristics between the ORC and RARC groups, 1:1 propensity score matching was performed using preoperative covariates, including the date of surgery and urinary diversion type (Figure 1). Overall survival (OS), cancer-specific survival (CSS), and recurrence-free survival (RFS) were compared between the two groups using Kaplan–Meier curves and the log-rank test. Univariable and multivariable Cox proportional hazards regression models were constructed to identify independent predictors of survival. The proportional hazards assumption was verified for all multivariable Cox models using Schoenfeld residuals, confirming that all variables satisfied the assumption (*p* value ≥ 0.05). Fine–Gray subdistribution hazards models were fitted as sensitivity analyses to account for competing risks when estimating the cumulative incidence of cancer-specific mortality. To further evaluate the robustness of our findings, we also performed inverse probability of treatment weighting in the entire cohort and conducted an additional sensitivity analysis using a secondary propensity score-matched cohort in which pathological T stage was explicitly matched.

All statistical analyses were conducted using IBM SPSS Statistics version 22.0 (IBM Corp., Armonk, NY, USA) and R version 4.3.2 (R Foundation for Statistical Computing, Vienna, Austria). A two-sided *p* value < 0.05 was considered to indicate statistical significance.

## 3. Results

### 3.1. Baseline Characteristics

Of 3972 patients initially assessed for eligibility, 1789 patients were included in the final cohort, of whom 1220 underwent ORC and 569 underwent RARC. Following 1:1 propensity score matching, 473 patients remained in each group (Figure 1). The baseline characteristics of the matched cohort were generally comparable, indicating that the two groups were well balanced after matching (Table 1).

### 3.2. Perioperative and Pathological Outcome

Perioperative and pathological outcomes are summarized in Table 2. As intended by the propensity score matching, the date of surgery and the type of urinary diversion were well balanced between the two groups. Compared with ORC, RARC was significantly associated with a longer operative time, reduced EBL, lower intraoperative transfusion rate, a shorter LOS, higher lymph node yields, and a lower incidence of positive STSMs (all *p* < 0.05). Also, the distribution of pathological T stage differed significantly, with the RARC group exhibiting a higher proportion of lower-stage tumors.

### 3.3. Kaplan–Meier Survival Analyses

For OS and CSS, the median follow-up lasted 40.2 months, while the median for RFS was 30.5 months. During this observation period, we recorded 283 total deaths (including 154 cancer-specific mortalities) and 354 instances of tumor recurrence. The estimated survival rates at 5 and 10 years were as follows: 65.8% and 56.8% for OS, 80.1% and 75.4% for CSS, and 57.3% and 49.1% for RFS, respectively.

**Figure 2:**
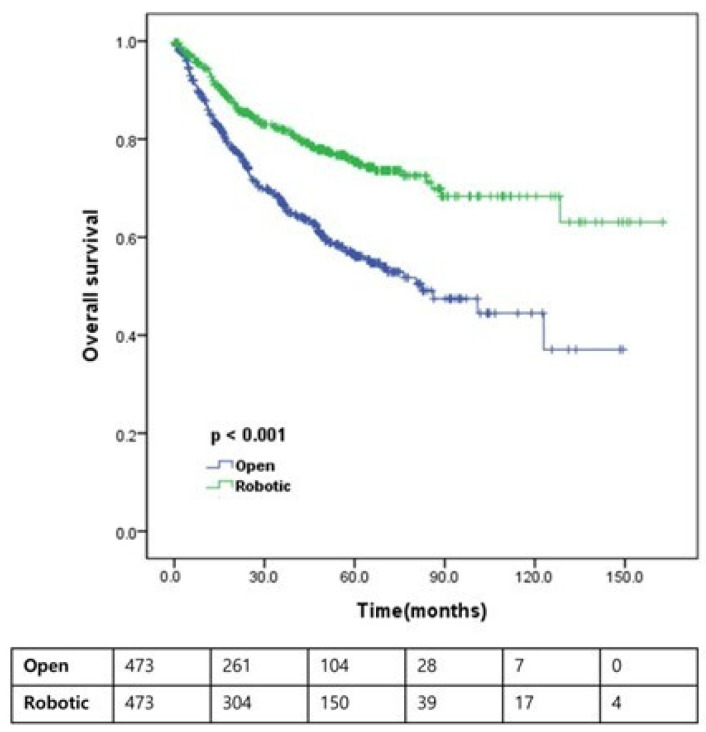
Overall survival according to the type of operation. Kaplan–Meier curves are presented with number at risk tables below the plot to improve readability and interpretation.

**Figure 3:**
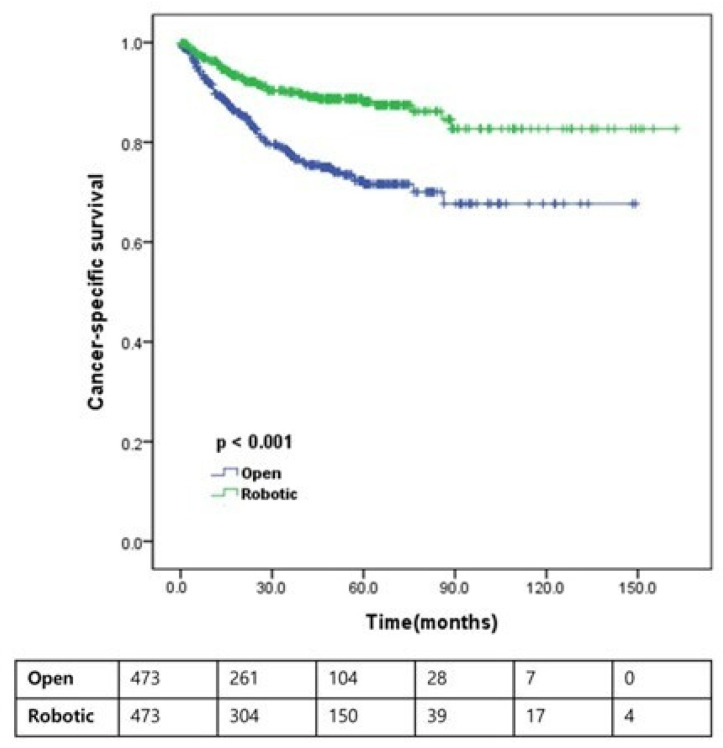
Cancer-specific survival according to the type of operation. Kaplan–Meier curves are presented with number at risk tables below the plot to improve readability and interpretation.

**Figure 4:**
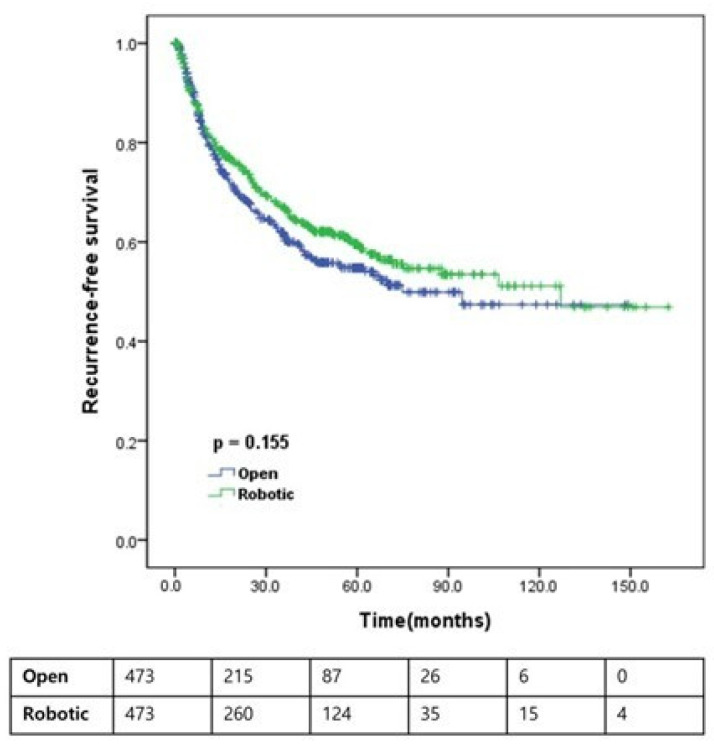
Recurrence-free survival according to the type of operation. Kaplan–Meier curves are presented with number at risk tables below the plot to improve readability and interpretation.

As illustrated in Figure 2, Figure 3 and Figure 4, Kaplan–Meier analyses demonstrated that RARC was significantly associated with improved OS (5-year: 75.4% vs. 56.1%; 10-year: 68.3% vs. 44.5%; *p* < 0.001) and CSS (5-year: 88.1% vs. 71.6%; 10-year: 82.7% vs. 67.7%; *p* < 0.001) compared with ORC. However, no significant difference was observed in RFS between the two approaches (5-year: 59.6% vs. 54.8%; 10-year: 51.1% vs. 47.4%; *p* = 0.155). While 10-year estimates are provided to illustrate long-term trends, these should be interpreted with caution given the reduced number of patients at risk at that time point.

### 3.4. Univariable and Multivariable Cox Proportional Hazards Analyses

#### 3.4.1. Overall Survival

Table 3 summarizes the univariable and multivariable Cox regression analyses for OS. In the univariable analysis, age, BMI, operation type, urinary diversion type, pathological T and N stages, lymph node yield, LVI, and STSM were significantly associated with OS. Multivariable analysis identified older age, higher pathological T and N stages, the presence of CIS, and LVI as independent predictors of worse OS. Importantly, RARC was independently associated with improved OS compared to ORC (hazard ratio [HR] 0.564; 95% confidence interval [CI] 0.440–0.722; *p* < 0.001).

#### 3.4.2. Cancer-Specific Survival

Table 4 summarizes the univariable and multivariable Cox regression analyses for CSS. In the univariable analysis, BMI, operation type, urinary diversion type, pathological T and N stages, LVI, and STSM were significantly associated with CSS. In the multivariable analysis, lower BMI, receipt of neoadjuvant CTx, higher pathological T and N stages, the presence of CIS, LVI, and STSM remained independently associated with worse CSS. Above all, RARC demonstrated a significant protective effect on CSS relative to ORC (HR 0.474; 95% CI 0.336–0.669; *p* < 0.001).

#### 3.4.3. Recurrence-Free Survival

Table 5 summarizes the univariable and multivariable Cox regression analyses for RFS. While concurrent UTUC, neoadjuvant CTx, urinary diversion type, pathological T and N stages, the presence of LVI, and STSM were significant in the univariable analysis, only higher pathological T and N stages remained independent predictors of worse RFS in the multivariable analysis. Moreover, the surgical approach was not an independent predictor of RFS, with no significant difference observed between RARC and ORC (HR 0.991; 95% CI 0.800–1.228; *p* = 0.936).

## 4. Discussion

This study compared survival outcomes between patients who underwent ORC and RARC utilizing a contemporary, large-scale, multicenter cohort balanced by propensity score matching. Our results demonstrate that RARC is associated with significantly better OS and CSS than ORC, whereas RFS remains comparable between the two approaches (Figure 2, Figure 3 and Figure 4). Notably, even after propensity score matching and multivariable adjustment for residual pathological imbalances—such as pathological T and N stages, lymph node yield, and STSM status—the robotic approach remained an independent favorable prognostic factor for both OS and CSS (Table 3 and Table 4). This finding was further corroborated by a supplementary sensitivity analysis utilizing a Fine–Gray competing-risk model for CSS (Table 6), where the surgical approach remained a significant predictor (HR 0.494; 95% CI 0.344–0.709; *p* < 0.001). The robustness of these results was further supported by an additional sensitivity analysis using inverse probability weighting, in which RARC consistently showed a significant survival benefit for both OS (HR = 0.810; *p* = 0.023) and CSS (HR = 0.818; *p* = 0.046) (Supplementary Table S1).

Historically, most randomized controlled trials (RCTs) and meta-analyses have demonstrated oncologic equivalence, rather than superiority, of RARC over ORC [13,14,15,16,17,18]. The RAZOR trial established non-inferior 2-year progression-free survival, reporting similar early recurrence and short-term survival rates for both cohorts [18]. Similarly, extended follow-up from another high-volume RCT revealed no significant differences in RFS, CSS, or OS between the approaches [19]. Systematic reviews pooling randomized and nonrandomized studies support these findings, concluding that OS and RFS are generally comparable while confirming lower transfusion rates, shorter length of stay, and longer operative times with RARC [15,16,20]. In this context, the survival advantage observed for RARC in the present study should be regarded as hypothesis-generating and interpreted cautiously.

Several factors may explain the survival benefit observed in our cohort. One plausible mechanism is that RARC was preferentially performed by more experienced surgeons in high-volume tertiary centers, thereby reflecting a synergy between the advanced surgical platform and concentrated surgical expertise. Previous studies have demonstrated a strong volume–outcome relationship in radical cystectomy, with higher hospital and surgeon volume associated with lower mortality, fewer complications, and improved long-term outcomes [21,22,23,24,25]. Furthermore, learning curve analyses indicate that as institutional experience with RARC accumulates, operative efficiency, lymph node yield, and perioperative outcomes concurrently improve [26,27,28,29]. Consequently, if high-volume or subspecialized surgeons were more likely to adopt and use the robotic platform, residual confounding by expertise could at least partially account for the apparent survival benefit, despite propensity matching and multivariable adjustment.

Residual selection bias stemming from unmeasured baseline patient and tumor characteristics presents another plausible explanation. In real-world clinical practice, surgical approach allocation is frequently influenced by nuanced factors such as frailty, subtle performance status variations, comorbidity burden, and socioeconomic status, which are rarely captured comprehensively in retrospective data [7,10,17]. Furthermore, surgeons may be more likely to choose ORC for patients presenting with higher perceived operative risks, anticipated technical difficulties, or more aggressive clinical phenotypes that are not adequately reflected by standard ASA scores or clinical staging [15,30]. Such unmeasured confounders inherently bias survival estimates against ORC, artificially inflating the comparative efficacy of RARC.

Potential mechanistic links between RARC and improved survival also warrant consideration. Meta-analyses consistently show that RARC is associated with lower blood loss and reduced transfusion rates compared to ORC [15,16,20]. Perioperative allogeneic blood transfusions have been independently linked to detrimental long-term oncologic outcomes across major cancer surgeries, potentially driven by transfusion-related immunomodulation and an increased complication burden [31,32]. Moreover, RARC is frequently associated with robust lymph node yields and equivalent, if not superior, negative margin rates—metrics inextricably linked to accurate staging and optimal cancer control [3,6,13,15]. In combination, these factors may contribute to improved CSS and OS in selected settings where the robotic approach is implemented by experienced surgical teams.

The discrepancy between improved OS/CSS and unchanged RFS in this study requires careful interpretation. One possibility is that the survival advantage may be driven less by a substantial reduction in cancer recurrence and more by a reduction in perioperative mortality, a better post-cystectomy health trajectory, and a higher tolerance for subsequent systemic therapies [33]. Randomized data suggest that RARC can reduce major complications and 90-day readmission without compromising oncologic endpoints, which may facilitate the timely delivery of systemic treatments and better recovery of functional status [18,19,34]. Additionally, institutional heterogeneity in surveillance protocols and recurrence definitions can obscure RFS differences, whereas mortality endpoints are often more reliably captured [13,17]. While our Fine–Gray competing-risk analysis strengthens the CSS findings by accounting for non-cancer-related deaths, it cannot entirely eliminate residual confounding.

Beyond survival, the perioperative findings of this study align well with the existing literature, highlighting lower EBL, decreased transfusion rates, and shorter LOS for RARC, juxtaposed with longer operative times [10,15,16,20]. The extended operative duration inherent to RARC is primarily attributable to technical prerequisites such as port placement, robotic docking, and the steep learning curve associated with robotic approach [6,13,18,19,30]. However, accumulating evidence indicates that prolonged operative times do not translate to elevated complication rates or inferior oncologic outcomes in RARC [26,27,28,29]. Therefore, the temporal drawback of the robotic approach should be weighed against its superior visualization, precise dissection capabilities, and favorable perioperative profile, which collectively offset the operative time penalty.

Several limitations of this study should be acknowledged. First, despite the use of propensity score matching and multivariable Cox regression, the retrospective design inherently limits the complete elimination of residual confounding. Specifically, the long study period (2003–2024) may introduce temporal bias as surgical techniques and perioperative care evolved considerably. Although we matched for the year of surgery and found that the majority of the cohort underwent surgery in recent years (2016–2019), it is possible that these adjustments do not fully capture the broader changes in clinical practice over two decades.

Second, we acknowledge the residual imbalance in pathological T stage distribution, with the robotic group exhibiting more favorable disease characteristics post-matching. Because pathological stage is a postoperative variable, including it in the primary matching could lead to over-matching and bias the results. However, recognizing that this stage distribution could plausibly influence survival estimates, we performed additional sensitivity analyses using inverse probability weighting and a cohort additionally matched for pT stage (Supplementary Tables S1 and S2). The consistency of the RARC survival advantage across these analyses suggests that the observed benefits are not merely a product of stage distribution, although a complete exclusion of this influence remains challenging.

Third, we must emphasize the absence of several critical confounders, such as surgeon experience, institutional volume, frailty, and socioeconomic status. As radical cystectomy is a complex procedure where surgeon and center volume significantly impact outcomes, the lack of these measures is a significant limitation. These unmeasured variables may provide alternative explanations for the observed differences between the ORC and RARC groups, and our findings should be interpreted with caution in this context.

Finally, our assessment of perioperative morbidity was limited to 90-day readmission as a crude surrogate, as standardized complication grades, such as the Clavien-Dindo classification, were unavailable due to the retrospective nature of the dataset. Consequently, the exact degree to which reduced perioperative morbidity mediated the survival advantage in the RARC group remains indeterminate. Additionally, the lack of central pathology review and variations in follow-up protocols across the 11 centers may have introduced inter-institutional heterogeneity. Since this cohort reflects tertiary referral practices in South Korea, the generalizability of these findings to more diverse global populations may be limited.

In conclusion, while this large-scale, multicenter analysis identified an association between RARC and improved OS and CSS, these preliminary observations must be interpreted with strict caution. The discrepancy between survival trends and RFS suggests that the observed outcomes may be influenced by residual confounding or factors inherent to high-volume centers rather than the surgical platform itself. Therefore, these findings should be viewed as hypothesis-generating, underscoring the need for prospective studies that can better isolate the impact of robotic technology from surgical expertise and patient selection.

## 5. Conclusions

In this large, multicenter, propensity score-matched cohort, RARC was associated with favorable perioperative outcomes and improved OS and CSS, while RFS remained comparable to ORC. However, given the potential for residual bias, these exploratory findings do not establish causal superiority and require a conservative interpretation. These results serve as a hypothesis-generating basis for future prospective trials.

## Figures and Tables

**Table 1:** Baseline characteristics of the study population.

Variables	ORC	RARC	*p* Value	SMD
Number of patients (*n*)	473	473	-	-
Age (year)			0.401	0.049
Mean (SD)	66.8 (10.1)	66.3 (10.3)		
Median (IQR)	68.0 (61.0–74.0)	67.0 (59.0–74.0)		
Sex			0.251	0.074
Men	416 (87.9)	404 (85.4)		
Women	57 (12.1)	69 (14.6)		
BMI (kg/m^2^)			0.457	0.037
Mean (SD)	24.00 (3.26)	24.12 (3.23)		
Median (IQR)	23.88 (22.01–25.79)	24.01 (22.05–26.11)		
HTN	237 (50.1)	221 (46.7)	0.298	0.068
DM	108 (22.8)	118 (24.9)	0.446	0.049
ASA status			0.976	0.016
1	48 (10.1)	50 (10.6)		
2	343 (72.5)	342 (72.3)		
≥3	82 (17.3)	81 (17.1)		
Clinical T stage			0.282	0.082
T < 2	123 (26.0)	139 (29.4)		
T2	152 (32.1)	131 (27.7)		
T3	151 (31.9)	164 (34.7)		
T4	47 (9.9)	39 (8.2)		
Clinical N stage			0.812	0.015
N0	430 (90.9)	428 (90.5)		
N1	26 (5.5)	30 (6.3)		
N2	13 (2.7)	13 (2.7)		
N3	4 (0 8)	2 (0.4)		
Concurrent UTUC	29 (6.1)	30 (6.3)	0.893	0.008
Neoadjuvant CTx	125 (26.4)	117 (24.7)	0.551	0.039

Data are presented as *n* (%) or as indicated in the table. ORC, open radical cystectomy; RARC, robot-assisted radical cystectomy; SMD, standardized mean difference; SD, standard deviation; IQR, interquartile range; BMI, body mass index; HTN, hypertension; DM, diabetes mellitus; ASA, American Society of Anesthesiologists; UTUC, upper tract urothelial carcinoma.

**Table 2:** Perioperative and pathological outcome of the study population.

Variables	ORC	RARC	*p* Value	SMD
Date of surgery (day/month/year)	30 October 2017 (3 March 2016–2 May 2019)	15 January 2018 (26 June 2016–2 May 2019)	0.334	0.046
Type of urinary diversion			0.432	0.063
Neobladder	234 (49.5)	248 (52.4)		
Ileal conduit	220 (46.5)	212 (44.8)		
Ureterocutaneostomy	19 (4.0)	13 (2.7)		
Operation time (min)	310 (250–407)	400 (325–486)	<0.001	0.674
EBL (mL)	750 (410–1225)	300 (300–500)	<0.001	0.862
Intraoperative transfusion	207 (43.8)	61 (12.9)	<0.001	0.730
LOS (days)	20 (15–28)	17 (14–22)	<0.001	0.303
Pathological T stage			0.010	0.193
T < 2	124 (26.2)	158 (33.4)		
T2	110 (23.3)	126 (26.6)		
T3	181 (38.3)	148 (31.3)		
T4	58 (12.3)	41 (8.7)		
No. LN removed			0.007	0.172
Mean (SD)	19 (10)	21 (13)		
Median (IQR)	17 (12–25)	19 (12–28)		
Pathological N stage			0.868	0.021
N0	348 (73.6)	345 (72.9)		
N1	46 (9.7)	53 (11.2)		
N2	67 (14.2)	65 (13.7)		
N3	12 (2.5)	10 (2.1)		
Grade			0.081	0.117
Low	22 (4.7)	12 (2.5)		
High	451 (95.3)	461 (97.5)		
LVI	189 (40.0)	167 (35.3)	0.140	0.097
Concurrent CIS	179 (37.8)	177 (37.4)	0.893	0.008
Positive STSM	48 (10.1)	30 (6.3)	0.033	0.138
90-day readmission	61 (12.9)	71 (15.0)	0.348	0.061
Adjuvant CTx	152 (32.1)	130 (27.5)	0.118	0.101

Data are presented as *n* (%) or median values (IQR) or as indicated in the table. ORC, open radical cystectomy; RARC, robot-assisted radical cystectomy; SMD, standardized mean difference; EBL; estimated blood loss; LOS, length of hospital stay; LN, lymph node; SD, standard deviation; IQR, Interquartile range; LVI, lymphovascular invasion; CIS, carcinoma in situ; STSM, soft tissue surgical margin; CTx, chemotherapy.

**Table 3:** Univariable and multivariable Cox proportional hazards analyses of overall survival.

Variables	Univariable		Multivariable	
	HR (95% CI)	*p* Value	HR (95% CI)	*p* Value
Age	1.030 (1.017–1.043)	<0.001	1.028 (1.014–1.043)	<0.001
Sex (male vs. female)	0.890 (0.627–1.263)	0.514	1.121 (0.783–1.604)	0.534
BMI	0.953 (0.917–0.990)	0.013	0.962 (0.923–1.001)	0.057
ASA (<3 vs. ≥3)	1.082 (0.771–1.519)	0.649	1.023 (0.725–1.444)	0.897
Concurrent UTUC (no vs. yes)	1.252 (0.795–1.974)	0.332	0.943 (0.582–1.529)	0.813
Neoadjuvant CTx (no vs. yes)	1.226 (0.944–1.593)	0.126	1.265 (0.968–1.653)	0.085
Operation type (open vs. robotic)	0.495 (0.389–0.631)	<0.001	0.564 (0.440–0.722)	<0.001
Type of urinary diversion		0.003		0.407
Neobladder	Ref	Ref	Ref	Ref
Ileal conduit	1.436 (1.130–1.824)	0.003	0.923 (0.706–1.206)	0.557
Ureterocutaneostomy	2.061 (1.139–3.729)	0.017	1.378 (0.723–2.623)	0.330
Pathological T stage		<0.001		<0.001
T < 2	Ref	Ref	Ref	Ref
T2	1.219 (0.815–1.823)	0.335	1.003 (0.659–1.525)	0.990
T3	3.230 (2.331–4.477)	<0.001	2.004 (1.365–2.943)	<0.001
T4	4.558 (3.063–6.783)	<0.001	2.443 (1.532–3.896)	<0.001
Pathological N stage (<1 vs. ≥1)	2.682 (2.109–3.386)	<0.001	1.801 (1.371–2.366)	<0.001
No. LN removed	0.987 (0.977–0.998)	0.019	0.992 (0.981–1.004)	0.184
Grade (low vs. high)	1.126 (0.616–2.058)	0.700	0.969 (0.526–1.788)	0.921
Concurrent CIS (no vs. yes)	1.177 (0.930–1.489)	0.175	1.380 (1.081–1.761)	0.010
LVI (no vs. yes)	2.538 (2.007–3.210)	<0.001	1.412 (1.058–1.883)	0.019
STSM (no vs. yes)	2.107 (1.478–3.003)	<0.001	1.444 (0.988–2.109)	0.058

HR, hazard ratio; CI, confidence interval; BMI, body mass index; ASA, American Society of Anesthesiologists; CTx, chemotherapy; UTUC, upper tract urothelial carcinoma; LN, lymph node; LVI, lymphovascular invasion; CIS, carcinoma in situ; STSM, soft tissue surgical margin.

**Table 4:** Univariable and multivariable Cox proportional hazards analyses of cancer-specific survival.

Variables	Univariable		Multivariable	
	HR (95% CI)	*p* Value	HR (95% CI)	*p* Value
Age	1.015 (0.998–1.031)	0.084	1.010 (0.992–1.028)	0.268
Sex (male vs. female)	0.713 (0.425–1.197)	0.201	0.915 (0.539–1.555)	0.744
BMI	0.922 (0.875–0.972)	0.003	0.926 (0.876–0.979)	0.007
ASA (<3 vs. ≥3)	1.315 (0.861–2.008)	0.205	1.317 (0.856–2.025)	0.211
Concurrent UTUC (no vs. yes)	1.489 (0.843–2.628)	0.170	1.043 (0.573–1.900)	0.890
Neoadjuvant CTx (no vs. yes)	1.379 (0.978–1.943)	0.067	1.422 (1.002–2.020)	0.049
Operation type (open vs. robotic)	0.422 (0.301–0.592)	<0.001	0.474 (0.336–0.669)	<0.001
Type of urinary diversion		0.013		0.100
Neobladder	Ref	Ref	Ref	Ref
Ileal conduit	1.303 (0.941–1.805)	0.111	0.852 (0.591–1.228)	0.390
Ureterocutaneostomy	2.693 (1.345–5.392)	0.005	1.829 (0.855–3.911)	0.120
Pathological T stage		<0.001		0.002
T < 2	Ref	Ref	Ref	Ref
T2	1.052 (0.592–1.870)	0.863	0.788 (0.432–1.439)	0.438
T3	3.134 (2.003–4.904)	<0.001	1.715 (1.006–2.923)	0.047
T4	5.505 (3.286–9.223)	<0.001	2.335 (1.249–4.364)	0.008
Pathological N stage (<1 vs. ≥1)	3.171 (2.308–4.357)	<0.001	2.007 (1.392–2.892)	<0.001
No. LN removed	0.989 (0.975–1.003)	0.137	0.993 (0.978–1.009)	0.397
Grade (low vs. high)	1.684 (0.624–4.548)	0.304	1.491 (0.543–4.090)	0.438
Concurrent CIS (no vs. yes)	1.217 (0.885–1.674)	0.227	1.457 (1.045–2.031)	0.026
LVI (no vs. yes)	2.967 (2.150–4.095)	<0.001	1.510 (1.013–2.252)	0.043
STSM (no vs. yes)	2.936 (1.924–4.481)	<0.001	1.962 (1.242–3.099)	0.004

HR, hazard ratio; CI, confidence interval; BMI, body mass index; ASA, American Society of Anesthesiologists; CTx, chemotherapy; UTUC, upper tract urothelial carcinoma; LN, lymph node; LVI, lymphovascular invasion; CIS, carcinoma in situ; STSM, soft tissue surgical margin.

**Table 5:** Univariable and multivariable Cox proportional hazards analyses of recurrence-free survival.

Variables	Univariable		Multivariable	
	HR (95% CI)	*p* Value	HR (95% CI)	*p* Value
Age	1.005 (0.994–1.015)	0.380	1.043 (0.754–1.442)	0.800
Sex (male vs. female)	0.872 (0.636–1.196)	0.396	1.043 (0.992–1.016)	0.541
BMI	0.975 (0.943–1.008)	0.136	0.985 (0.951–1.020)	0.395
ASA (<3 vs. ≥3)	1.089 (0.815–1.457)	0.563	0.996 (0.741–1.339)	0.979
Concurrent UTUC (no vs. yes)	1.514 (1.035–2.214)	0.032	1.269 (0.851–1.893)	0.243
Neoadjuvant CTx (no vs. yes)	1.276 (1.010–1.613)	0.041	1.244 (0.977–1.584)	0.077
Operation type (open vs. robotic)	0.860 (0.698–1.059)	0.156	0.991 (0.800–1.228)	0.936
Type of urinary diversion		0.026		0.833
Neobladder	Ref	Ref	Ref	Ref
Ileal conduit	1.335 (1.081–1.649)	0.007	1.049 (0.826–1.332)	0.696
Ureterocutaneostomy	1.040 (0.532–2.035)	0.908	0.879 (0.434–1.781)	0.721
Pathological T stage		<0.001		<0.001
T < 2	Ref	Ref	Ref	Ref
T2	1.766 (1.240–2.515)	0.002	1.501 (1.038–2.171)	0.031
T3	3.932 (2.896–5.339)	<0.001	2.610 (1.830–3.723)	<0.001
T4	6.250 (4.344–8.992)	<0.001	3.616 (2.373–5.508)	<0.001
Pathological N stage (<1 vs. ≥1)	3.313 (2.679–4.096)	<0.001	2.041 (1.597–2.608)	<0.001
No. LN removed	0.995 (0.987–1.005)	0.327	0.996 (0.987–1.006)	0.475
Grade (low vs. high)	1.045 (0.612–1.783)	0.872	0.849 (0.493–1.464)	0.556
Concurrent CIS (no vs. yes)	0.960 (0.775–1.190)	0.712	1.152 (0.923–1.438)	0.211
LVI (no vs. yes)	2.672 (2.166–3.296)	<0.001	1.283 (0.992–1.658)	0.058
STSM (no vs. yes)	1.968 (1.421–2.726)	<0.001	1.298 (0.919–1.832)	0.139

HR, hazard ratio; CI, confidence interval; BMI, body mass index; ASA, American Society of Anesthesiologists; CTx, chemotherapy; UTUC, upper tract urothelial carcinoma; LN, lymph node; LVI, lymphovascular invasion; CIS, carcinoma in situ; STSM, soft tissue surgical margin.

**Table 6:** Univariable and multivariable competing-risk regression analyses of cancer-specific survival.

Variables	Univariable		Multivariable	
	HR (95% CI)	*p* Value	HR (95% CI)	*p* Value
Age	1.010 (0.992–1.029)	0.280	1.005 (0.986–1.025)	0.600
Sex (male vs. female)	0.705 (0.422–1.178)	0.180	0.903 (0.540–1.512)	0.700
BMI	0.927 (0.877–0.979)	0.007	0.929 (0.876–0.986)	0.015
ASA (<3 vs. ≥3)	1.302 (0.847–2.001)	0.230	1.342 (0.857–2.101)	0.200
Concurrent UTUC (no vs. yes)	1.450 (0.836–2.515)	0.190	1.088 (0.578–2.046)	0.790
Neoadjuvant CTx (no vs. yes)	1.371 (0.974–1.930)	0.071	1.385 (0.978–1.961)	0.066
Operation type (open vs. robotic)	0.440 (0.314–0.616)	<0.001	0.494 (0.344–0.709)	<0.001
Type of urinary diversion		0.029		0.608
Neobladder	Ref	Ref	Ref	Ref
Ileal conduit	1.249 (0.902–1.730)	0.180	0.903 (0.630–1.297)	0.580
Ureterocutaneostomy	2.516 (1.236–5.120)	0.011	1.357 (0.543–3.395)	0.510
Pathological T stage		<0.001		0.007
T < 2	Ref	Ref	Ref	Ref
T2	1.032 (0.582–1.831)	0.910	0.831 (0.466–1.483)	0.530
T3	2.838 (1.818–4.430)	<0.001	1.691 (0.978–2.925)	0.060
T4	4.995 (2.994–8.335)	<0.001	2.327 (1.215–4.458)	0.011
Pathological N stage (<1 vs. ≥1)	2.909 (2.120–3.990)	<0.001	1.822 (1.255–2.644)	0.002
No. LN removed	0.991 (0.975–1.007)	0.270	0.997 (0.981–1.013)	0.730
Grade (low vs. high)	1.660 (0.629–4.386)	0.310	1.448 (0.550–3.810)	0.450
Concurrent CIS (no vs. yes)	1.207 (0.879–1.656)	0.240	1.474 (1.053–2.062)	0.024
LVI (no vs. yes)	2.758 (2.000–3.803)	<0.001	1.396 (0.909–2.143)	0.130
STSM (no vs. yes)	2.823 (1.848–4.313)	<0.001	1.854 (1.129–3.044)	0.015

HR, hazard ratio; CI, confidence interval; BMI, body mass index; ASA, American Society of Anesthesiologists; CTx, chemotherapy; UTUC, upper tract urothelial carcinoma; LN, lymph node; LVI, lymphovascular invasion; CIS, carcinoma in situ; STSM, soft tissue surgical margin.

## Data Availability

The data presented in this study are available on reasonable request from the corresponding author. The data are not publicly available due to patient privacy considerations.

## Supplementary Materials

The following supporting information can be downloaded at: https://www.mdpi.com/article/10.3390/jcm15093559/s1.

## Abbreviations

The following abbreviations are used in this manuscript:

BC	Bladder cancer
RC	Radical cystectomy
PLND	Pelvic lymph node dissection
RARC	Robot-assisted radical cystectomy
ORC	Open radical cystectomy
UTUC	Upper tract urothelial carcinoma
HTN	Hypertension
DM	Diabetes mellitus
BMI	Body mass index
ASA	American Society of Anesthesiologists
CTx	Chemotherapy
EBL	Estimated blood loss
LOS	Length of hospital stay
STSM	Soft tissue surgical margins
CIS	Carcinoma in situ
LVI	Lymphovascular invasion
SD	Standard deviation
IQR	Interquartile range
OS	Overall survival
CSS	Cancer-specific survival
RFS	Recurrence-free survival
HR	Hazard ratio
CI	Confidence interval
