# Supplementary material for: Survival Outcomes of Open Versus Robot-Assisted Radical Cystectomy: A Large-Scale Multicenter Propensity Score Matched Study
Source: J Clin Med. 2026 May 6;15(9):3559. doi: 10.3390/jcm15093559 (PMC13163848; doi:10.3390/jcm15093559)
Supplement: Supplementary file 1 [file jcm-15-03559-s001.zip › Supplementary Table S1.pdf]

Supplementary Table S1. Multivariable cox proportional hazards analyses for overall and cancer-specific survival in the inverse probability of treatment weighted cohort

Variables	Overall survival		Cancer-specific survival	
	HR (95% CI)	P value	HR (95% CI)	P value
Age	1.029 (1.014 – 1.044)	< 0.001	1.015 (0.999 – 1.031)	0.066
Sex (male vs female)	1.251 (0.874 – 1.791)	0.221	1.951 (0.680 – 1.765)	0.709
BMI	0.956 (1.014 – 1.044)	0.037	0.960 (0.9112 – 1.011)	0.119
ASA (<3 vs ≥3)	1.209 (0.891 – 1.639)	0.223	1.360 (0.964 – 1.919)	0.080
Concurrent UTUC (no vs yes)	0.977 (0.632 – 1.510)	0.917	0.878 (0.526 – 1.468)	0.621
Neoadjuvant CTx (no vs yes)	1.878 (1.468 – 2.403)	< 0.001	1.691 (1.232 – 2.319)	0.001
Operation type (Open vs Robotic)	0.810 (0.676 – 0.971)	0.023	0.818 (0.671 – 0.997)	0.046
Type of urinary diversion		0.816		0.858
Neobladder	Ref	Ref	Ref	Ref
Ileal conduit	0.913 (0.685 – 1.217)	0.536	1.002 (0.696 – 1.437)	0.992
Ureterocutaneostomy	0.959 (0.603 – 1.524)	0.859	1.138 (0.664 – 1.949)	0.638
Pathological T stage		< 0.001		0.003
T < 2	Ref	Ref	Ref	Ref
T2	0.766 (0.493 – 1.189)	0.234	0.784 (0.461- 1.333)	0.369
T3	1.642 (1.128 – 2.390)	0.010	1.688 (1.064 – 2.677)	0.026
T4	1.702 (1.147 – 2.526)	0.008	1.682 (1.033 – 2.738)	0.037
Pathological N stage (<1 vs ≥1)	1.950 (1.447 – 2.626)	< 0.001	1.982 (1.400 – 2.808)	< 0.001
No. LN removed	0.982 (0.969 – 0.996)	0.013	0.976 (0.959 – 0.994)	0.010
Grade (Low vs High)	1.015 (0.553 – 1.862)	0.962	2.382 (0.861- 1.255)	0.094
Concurrent CIS (no vs yes)	0.917 (0.706 – 1.193)	0.521	0.924 (0.680 – 1.255)	0.612
LVI (no vs yes)	1.269 (0.964 – 1.670)	0.089	1.300 (0.927 – 1.823)	0.129
STSM (no vs yes)	1.542 (1.111 – 2.140)	0.010	1.758 (1.227 – 2.519)	0.002

HR, hazard ratio; CI, confidence interval; BMI, Body mass index; ASA, American Society of Anesthesiologists; CTx, Chemotherapy; UTUC, Upper tract urothelial carcinoma; LN, lymph node; LVI, lymphovascular invasion; CIS, carcinoma in situ; STSM, soft tissue surgical margin.
