# Supplementary material for: Survival Outcomes of Open Versus Robot-Assisted Radical Cystectomy: A Large-Scale Multicenter Propensity Score Matched Study
Source: J Clin Med. 2026 May 6;15(9):3559. doi: 10.3390/jcm15093559 (PMC13163848; doi:10.3390/jcm15093559)
Supplement: Supplementary file 1 [file jcm-15-03559-s001.zip › Supplementary Table S2.pdf]

Supplementary Table S2. Multivariable cox proportional hazards analyses for overall and cancer-specific survival in the pathological T stage–matched cohort

Variables	Overall survival		Cancer-specific survival	
	HR (95% CI)	P value	HR (95% CI)	P value
Age	1.025 (1.010 - 1.025)	0.001	1.011 (0.992 – 1.030)	0.250
Sex (male vs female)	1.234 (0.864 - 1.762)	0.247	1.048 (0.617 – 1.783)	0.862
BMI	0.965 (0.927 – 1.005)	0.089	0.937 (0.885 – 0.992)	0.026
ASA (<3 vs ≥3)	0.859 (0.600 – 1.231)	0.409	0.959 (0.604 – 1.523)	0.860
Concurrent UTUC (no vs yes)	1.192 (0.756 – 1.878)	0.449	1.126 (0.623 – 2.037)	0.694
Neoadjuvant CTx (no vs yes)	1.192 (1.014 – 1.723)	0.039	1.462 (1.024 – 2.086)	0.037
Operation type (Open vs Robotic)	0.661 (0.517 – 0.843)	0.001	0.537 (0.383 – 0.753)	< 0.001
Type of urinary diversion		0.680		0.063
Neobladder	Ref	Ref	Ref	Ref
Ileal conduit	1.049 (0.802 – 1.372)	0.727	0.960 (0.664 – 1.387)	0.827
Ureterocutaneostomy	1.348 (0.687 – 2.643)	0.385	2.290 (1.083 – 4.845)	0.030
Pathological T stage		< 0.001		0.009
T < 2	Ref	Ref	Ref	Ref
T2	1.137 (0.773 – 1.675)	0.514	0.988 (0.572 – 1.707)	0.965
T3	2.174 (1.521 – 3.108)	< 0.001	1.954 (1.188 – 3.214)	0.008
T4	2.235 (1.360 – 3.674)	0.002	1.780 (0.909 – 3.484)	0.092
Pathological N stage (<1 vs ≥1)	1.825 (1.381 – 2.413)	< 0.001	2.275 (1.566 – 3.305)	< 0.001
No. LN removed	0.990 (0.978 – 1.001)	0.087	0.986 (0.970 – 1.002)	0.092
Grade (Low vs High)	0.986 (0.589 – 1.650)	0.957	2.997 (0.944 – 9.518)	0.063
Concurrent CIS (no vs yes)	1.435 (1.121 – 1.836)	0.004	1.446 (1.029 – 2.030)	0.033
LVI (no vs yes)	1.217 (0.920 – 1.609)	0.169	1.205 (0.827 – 1.756)	0.332
STSM (no vs yes)	1.268 (0.836 – 1.925)	0.264	1.869 (1.137 – 3.070)	0.014

HR, hazard ratio; CI, confidence interval; BMI, Body mass index; ASA, American Society of Anesthesiologists; CTx, Chemotherapy; UTUC, Upper tract urothelial carcinoma; LN, lymph node; LVI, lymphovascular invasion; CIS, carcinoma in situ; STSM, soft tissue surgical margin.
